# Investigation into the controversial association of *Streptococcus gallolyticus *with colorectal cancer and adenoma

**DOI:** 10.1186/1471-2407-9-403

**Published:** 2009-11-19

**Authors:** Ahmed S Abdulamir, Rand R Hafidh, Layla K Mahdi, Tarik Al-jeboori, Fatimah Abubaker

**Affiliations:** 1 Microbiology Research Department, Faculty of Medicine, University Putra Malaysia, 43400, UPM, Serdang, Selangor Darul Ehsan, Malaysia; 2 School of Molecular and Biomedical Science, University of Adelaide, South Australia, 5005; 3 Medical Microbiology Department, College of Medicine, Alnahrain University, 14222, Iraq; 4 Institute of bioscience, University Putra Malaysia, 43400, UPM, Serdang, Selangor Darul Ehsan, Malaysia

## Abstract

**Background:**

The seroprevalence of IgG antibodies of *Streptococcus gallolyticus subspecies gallolyticus*, CIP 105428, was evaluated to investigate the controversial association of *S. gallolyticus *with colorectal carcinoma and adenoma in attempt to investigate the nature of such association if any, by exploring the mRNA expression of NF-κB and IL-8. Moreover, the serological behavior of *S. gallolyticus *IgG antibodies was compared to that of an indicator bacterium of bowel, *Bacteroides fragilis*.

**Methods:**

ELISA was used to measure IgG antibodies of *S. gallolyticus *and *B. fragilis *in sera of 50 colorectal cancer, 14 colorectal adenoma patients, 30 age- and sex- matched apparently healthy volunteers (HV) and 30 age- and sex- matched colonoscopically-proven tumor-free control subjects. NF-κB and IL-8 mRNA expression was evaluated in tumorous and non-tumorous tissue sections of carcinoma and adenoma patients in comparison with that of control subjects by using in situ hybridization assay.

**Results:**

Colorectal cancer and adenoma patients were associated with higher levels of serum *S. gallolyticus *IgG antibodies in comparison with HV and control subjects (P < 0.05) while no similar association was found with serum IgG antibodies of *B. fragilis *(P > 0.05). ELISA cutoff value for the seropositivity of *S. gallolyticus *IgG was calculated from tumor-free control group. The expression of NF-κB mRNA was higher in tumorous than non-tumorous tissue sections of adenoma and carcinoma, higher in carcinoma/adenoma sections than in control subjects, higher in tumorous sections of carcinoma than in adenoma patients, and higher in *S. gallolyticus *IgG seropositive than in seronegative groups in both tumorous and non-tumorous sections (P < 0.05). IL-8 mRNA expression in tumorous sections of adenoma and carcinoma was higher than in non-tumorous sections, higher in carcinoma/adenoma than in control subjects, and higher in *S. gallolyticus *IgG seropositive than in seronegative groups in tumorous rather than non-tumorous sections (P < 0.05).

**Conclusion:**

*S. gallolyticus *most likely plays an essential role in the oncogenic progression of normal colorectal mucosa to adenoma and to CRC. This promoting/propagating role of *S. gallolyticus *might take place by utilizing certain inflammatory, anti-apoptotic, and angiogenic factors of transformation including NF-κB and IL-8.

## Background

Colorectal cancer (CRC) is the fourth commonest form of cancer occurring worldwide. The number of new cases of colorectal cancer has been increasing rapidly since 1975 [1]. Several studies have associated bacterial infections to carcinogenesis [2,3]. CRC was associated with Streptococcus bovis (*S. bovis*); the incidence of the association of colonic neoplasia with *S. bovis *has been determined as 18% to 62% [4,5]. Colonic neoplasia may arise years after the presentation of the condition of bacteremia or infectious endocarditis of *S. bovis *[5,6].

Prior to the description of *S. gallolyticus*, it was reported that among *S. bovis *biotypes identified by the API Rapid Strep system and cellular fatty acid content, biotype I was more likely than biotype II to be associated with both endocarditis and malignant or premalignant colonic lesion [7]. Following the description of *S. gallolyticus*, Devriese team showed that the bacterial isolates, which were studied previously and derived from patients with endocarditis and associated with colonic cancers and identified by conventional techniques as *S. bovis*, were in fact *S. gallolyticus *[8]. They suggested that *S. gallolyticus *is more likely to be involved in human infections than *S. bovis *and most of *S. gallolyticus *strains belong to the so-called *S. bovis *biotype I and a few belong to *S. bovis *biotype II/2. Recently *S. gallolyticus *subspecies *gallolyticus *has become the most implicated agent in the association with CRC as Schlegel et al. stated that most of the human strains isolated from blood or feces were *Streptococcus gallolyticus *which is often responsible for endocarditis cases associated with a colonic cancer [9].

After the new species, *S. gallolyticus*, was assigned, there has been no specific serological study done for the association between *S. gallolyticus *and CRC or colorectal adenoma. Therefore, we conducted a serological investigation of *S. gallolyticus *IgG antibodies in CRC and colorectal adenoma patients in comparison with normal individuals. To keep the scientific fidelity, we accompanied another intestinal bacterium, namely *Bacteroides fragilis *(*B. fragilis*), strain ATCC 25285. *B. fragilis *is one of the most dominant bacteria in the normal flora of humans' large intestine and present in bowel at incidence of 100% [10]. *B. fragilis *was selected for this comparison because *B. fragilis *is confined to the bowel and isolated from the blood circulation by an integral mucosal barrier; any breach, say degenerative lesion or ulceration, in the mucosal barrier of the bowel leads to showering of huge amount of *B. fragilis *into blood circulation which results in a vigorous immune response [11]. Although no quantitative comparison was aimed between the seroprevalence of *B. fragilis *and *S. gallolyticus*, as they are of different species, we intended to compare the behavior or trend of the seroprevalence of *B. fragilis *lipopolysaccharides (LPS) IgG antibodies among CRC, adenoma and normal subjects to that of our target bacteria, *S. gallolyticus *cell wall antigens IgG antibodies. The reason behind that was to assess whether the association of *S. gallolyticus *with CRC and adenoma is specific or other bowel bacteria are associated too through a non-specific entry of bowel bacteria into blood circulation via tumor lesion. In addition, LPS of *B. fragilis *is a specific antigen and not shared with other Gram negative/positive bacteria. It was reported that no cross-reactivity between *B. fragilis *LPS and LPS of other bacteria was observed and *B. fragilis *LPS contains an immunodominant antigenic determinant common to almost all *B. fragilis *isolates [11,12]. Moreover, it was revealed that anti-*B. fragilis *IgM, IgG, and IgA antibodies in mice and human serum were measured reliably by using *B. fragilis *LPS in enzyme immune assays [13,14].

Previous experiments by our team showed no link between *S. gallolyticus*-associated CRC and a number of tumor suppressor proteins, p53, p21, and p27 [unpublished data] as these factors were found to be greatly associated with the transformation process of colorectal mucosa to adenoma and then to carcinoma [15]. Therefore, in the current study, un-researched before, target molecules, namely, nuclear factor kappa-B (NF-κB) and interleukin-8 (IL-8) were studied in relation with *S. gallolyticus *as well as the transformation of colorectal mucosa to adenoma and carcinoma. Accordingly, the mRNA expression of NF-κB, a central transcriptional factor for inflammation [16] and IL-8, a powerful angiogenic chemokine which is frequently related to bacterial carcinogenesis e.g. *H. pylori *[17], were evaluated using in situ hybridization (ISH) assay.

## Methods

### The population of the study

CRC patients were those who attended several Gastroenterology and Hepatology Centers in the state of Selangor, Malaysia from March 2006 to December 2007, that underwent elective surgical resection of colorectal cancer. Adenoma patients were those who were referred to do colonoscopy due to various reasons and resection of intestinal polyps was done. Twenty seven men and 23 women with primary colorectal adenocarcinoma were included in this study prior to any chemotherapy. On the other hand, seven men and seven women with colorectal adenoma who had already undergone colonoscopical resection of adenomatous polyps were involved in this study. The medical history of the involved patients in this study was evaluated; no one had history of gastrointestinal disease or ulceration which might affect the seroprevalence of *S. gallolyticus *or *B. fragilis*. On the other hand, 30 age- and sex- matched control subjects were involved. They were referred to hospitals for doing colonoscopy for various reasons in whom normal colonic mucosa was confirmed and no other gastrointestinal disease or history of gastrointestinal diseases and ulcerations were found. Colonoscopical biopsy was taken from control subjects to compare the level of IL-8 and NFKB mRNA expression with that in mucosal colonic tissues from patients with adenoma and CRC. Moreover, blood samples were taken from the control group to calculate the seropositivity cutoff value for *S. gallolyticus *IgG antibodies. The resultant cutoff value was applied on CRC and adenoma patients as well as on age- and sex- matched 30 apparently healthy volunteers group (HV) who agreed to do colonoscopy in case results of ELISA would show high level of *S. gallolyticus *IgG antibodies. HV were medically examined and their medical records and history were reviewed and no major or gastrointestinal illness was found. Written consents were officially obtained from all participants in this study. The study was carried out in the scope of Helsinki declaration of ethical principles of medical research and permission was granted from the Ethics Committee of biomedical research of University Putra Malaysia.

### Sampling and processing of specimens

Samples of 3 to 5 ml of blood for serum isolation were withdrawn from control and adenoma groups at time of colonoscopy, from HV group after taking the medical history, and from CRC patients 2-3 days before surgery. Regarding the histopathology, a set of steps was pursued under the supervision of a pathologist to minimize as could as possible the fixation-related loss of mRNA. These steps were minimal prefixation time of 1 hour, the use of cold 4% paraformaldehyde, cold fixation at 4°C, and short duration of fixation, up to 5 hours [18]. It was stated that no significant loss of nucleic acids was observed within the first 3 days of fixation-paraffin embedding [19]. Therefore, paraffin-embedded sections were processed for ISH examination in a period of not more than 3 days. Moreover, the scoring system used in this study did not rely on the quantitative measurement of staining intensity. Therefore, mRNA loss, which affects mainly the intensity of staining, was believed to affect the results of this study minimally. To evaluate the stringent conditions pursued for minimizing mRNA loss, the ISH immunostaining was compared between 6 randomly selected paraffin embedded sections and cryostat sections. Despite the bit lower intensity of staining, the mean percentage of the positively stained cells was not changed. Hence it was confirmed that no loss of mRNA took place which could affect ISH results. Each histopathological paraffin block of excisional biopsies of CRC patients and punch biopsies of control subjects and adenoma patients were sectioned into 4 um thick sections. Histopathological sections were made from both tumorous and non-tumorous tissues for each CRC (resection safe margins) and adenoma patient (punch biopsy 3-4 cm away from the polyp). Hematoxylin and Eosin slides were prepared and examined by a histopathologist for confirming the histopathological diagnosis, the grade of CRC, and the type and degree of dysplasia of adenoma tissue sections.

### Extraction of cell wall antigens of *S. gallolyticus *and LPS of *B. fragilis*

The reference strain, *S. gallolyticus *subspecies *gallolyticus *CIP 105428 (Insitut de Louis Pasteur, France) was used. The extraction of cell wall antigens of *S. gallolyticus *was conducted by using the lysozyme method. Sufficient amount of the cultured reference bacteria in Columbia agar (Oxoid, UK) with 5% horse blood was obtained by making bacterial suspension in 30 mM (pH 8) Tris buffer (Fluka, Switzerland). The suspension was centrifuged at 3500 g for 10 minutes at 4°C. Pellet was washed 3 times in 30 mM Tris buffer (pH 8) by centrifugation at 3500 g for 5 minutes at 4°C. Pellet of washed bacteria was resuspended in a solution containing 4.75 ml of 30 mM Tris buffer (pH 8), 3 mM magnesium chloride (Sigma, USA), 25% sucrose (Fluka, Switzerland) and 0.25 ml of lysozyme (0.6 mg/ml) (Sigma, USA). The lysate solution was incubated for 2 hours at 37°C and was then centrifuged at 3000 g for 5 minutes at 4°C. Then, the supernatant was collected, which became the solution of cell wall antigens of the reference strain, *S. gallolyticus *[20]. The concentration of cell wall antigens was measured by Biurette method.

The extraction of *B. fragilis *LPS was obtained by the phenol-water extraction method, followed by the phenol-chloroform-petroleum ether extraction, as described by [21].

### ELISA

For *S. gallolyticus*, 96-wells microtiter plate (Sterilin, UK) was coated with 40 μg/ml of cell wall antigen while, for *B. fragilis*, the microtiter plate was coated with 10 μg/ml of LPS extract. The concentration of coating antigens for the studied bacteria was determined after a series of standardization steps. The microtiter plates were incubated in a humid chamber for 2 hours at 37°C and were then washed and stored at -20°C until further use. It is noteworthy to mention that the cell wall antigens of *S. gallolyticus *had already been treated with 100 μl a well of 0.01 M sodium periodate (Sigma, USA) in PBS for two hours at room temperature to destroy the common polysaccharides antigen of group D. After 3 times washing, 50 μl sera of CRC (50), adenoma (14), control subjects (30), and healthy volunteers (30) were pipetted into microtiter plates and incubated for 2 hours at room temperature. For each run, two wells were dispensed with 50 μl of diluting buffer as a negative control and two wells were dispensed with known positive sera for either *S. gallolyticus *or *B. fragilis*. After 3 rounds of washing, 50 μl/well of 1:40,000 diluted horseradish peroxidase anti-human IgG conjugates (Sigma, USA) were dispensed and incubated for 2 hours at room temperature. Then, 50 μl/well of chromogen-substrate OPD.2HCL (Abbott, USA) were pipetted into wells and incubated in dark for 15 minutes at room temperature. Optical density was read by ELISA reader (Organic Technica, Spain) at 492 nm [22].

### Calculation of the cutoff value for *S. gallolyticus *seropositivity

The cut off value is considered as the upper limit above which all of readings are considered positive. ELISA readings of control subjects (n = 30) were used to calculate the cutoff value according to the following formula [23]:

[23] (2.462): taken from the table of student's *t*-test under the *P *= 0.01 for the 29 degrees of freedom.

The cutoff value was used to demarcate between the S. gallolyticus- seropositive and seronegative subjects in the participants of this study other than control group, namely CRC, adenoma, and HV groups

### In situ hybridization assay

For each run of ISH, one negative control tissue section (diluting buffer instead of probes), one positive tissue section (already tested as strongly positive), and one endogenous positive probe control were used. Biotinylated long DNA probe for human NF-κB mRNA and human IL-8 mRNA were used (Maximbio, USA). The used procedure was according to "DNA probe Hybridization/Detection System - in situ Kit" (Maximbio, USA).

Slides were baked overnight at 70°C and deparaffinized in xylene (Merck, Germany) and descending grades of ethanol (Merck, Germany) starting from 100%. Freshly diluted 1× proteinase K (Sigma, USA) solution was applied for 15 min at 37°C. The working solutions used for probes were 10% v/v for NF-κB and 7% v/v for IL-8 mRNA. Ten μl of the working cDNA probe was added onto each slide. Slides were incubated in a humid chamber overnight at 37°C. Next day after washing slides, RNase A (Maximbio, USA) was added for 30 min to abolish any unbound RNA. Slides were then washed three times with a pre-warmed protein block for 3 minutes at 37°C. One to two drops of alkaline phosphatase-streptavidin conjugate (Abbott, USA) were added onto tissue sections for 1.5 hour at 37°C. Then, 1-2 drops of NBT/BCIP (Abbott, USA) substrate were placed on tissue sections at 37°C until color was developed. Dark blue colored precipitate was seen in positive cells. Slides were counterstained by nuclear fast red, dehydrated by graded alcohols, and mounted with a permanent-mounting DPX medium.

### Staining analysis

The scoring systems for in situ hybridization staining of NF-κB and IL-8 mRNA calculate the percentage of the glandular mucosal (adeno) cells, which were stained with nuclear blue/black color, out of total cells in 5 high power fields. The scores for NF-κB mRNA staining are; negative for less than 5% staining, low for 5-25% staining, intermediate for 26-50% staining, and positive for more than 50% staining [24]. The scores for IL-8 mRNA are; score 1 for 1-10% staining, score 2 for 11-50% staining, and score 3 for 51-100% staining [25]. In addition, stromal cells rather than glandular mucosal cells were observed too. The percentage of IL-8- or NF-κB- positively stained stromal cells were calculated out of total cells in 5 high power fields. However, no specific scoring system was found for stromal cells of human colonic tissues. Therefore, the ISH staining percentage of stromal cells was used for comparisons without using a predetermined scoring system.

### Statistical analysis

SPSS software version 12 for Windows (SPSS Inc., USA) and Excel XP (Microsoft, USA) were used. To validate the scoring systems used in this study, two sets of statistical analyses were used. The first set, Chi square for independence and Mann-Whitney tests were used for the scoring systems of NF-κB and IL-8 respectively. The second set, a univariate student *t *test was used for direct comparisons of the mean percentages of the positively stained cells. On the other hand for stromal cells, only student *t *test for mean percentages of the positively stained cells was used. Pearson's correlation coefficient was used to correlate the expression of IL-8 mRNA and NF-κB mRNA with *S. gallolyticus *IgG antibodies in both CRC and adenoma patients. P values less than 0.05 were considered significant.

## Results

### Demographic and histopathological features of CRC and adenoma patients

The mean age of CRC and adenoma patients was 57.08 years, ranged between 43 and 76 years, and 50.1 years, ranged between 37 and 70 years, respectively. On the other hand, the mean age of control and HV groups was 53.5 years, ranged between 38 and 78 years, and 52.8 years, ranged between 41 and 73 years, respectively. The whole of CRC cases were of adenocarcinoma; 32 cases were left-sided, 12 right-sided, and 6 at transverse colon. Seven out of 32 left-sided CRC were located in the rectum. Three (6%) of CRC patients were presented at B1, 5 (10%) at B2, 5 (10%) at C1, 7 (14%) at C2, and 30 (60%) at D stages. It was found that 30 (60%) of histological sections of CRC patients were poorly differentiated and 20 (40%) were mild-moderate differentiated. For adenoma, it was found that 6 patients (43%) were of villous type, 5 (35.7%) were of tubulovillous type, and only 3 (21.4%) were of tubular adenomatous polyps.

### Seroprevalence of IgG antibodies for *S. gallolyticus *and *B. fragilis*

**Figure 1:**
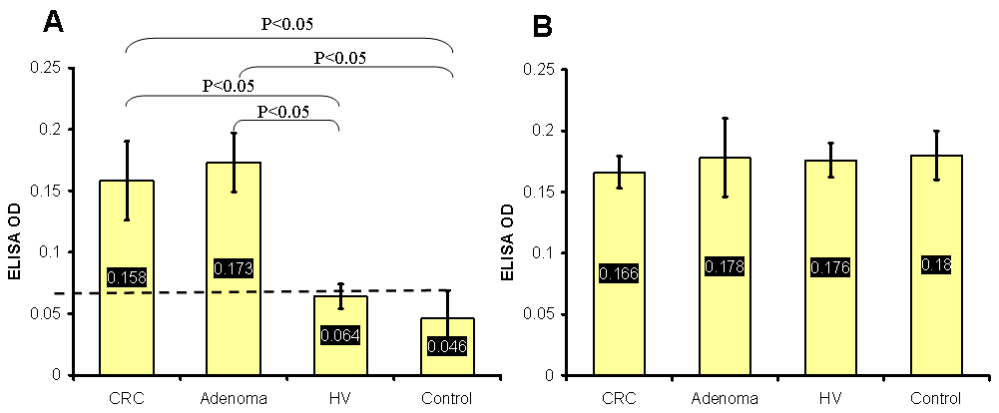
**Serum levels of IgG antibodies**. (A) The serum level of IgG antibodies of *S. gallolyticus *in terms of ELISA OD values. The high level of serum IgG antibodies of *S. gallolyticus *was detected in both CRC and adenoma patients which were significantly higher than that of HV and control group. The dashed line represents the ELISA cutoff value (OD 0.07) for the *S. gallolyticus *seropositivity measured from the tumor-free control group and applied on CRC, adenoma and HV groups. (B) The serum level of IgG antibodies of *B. fragilis*, an indicator bacterium for comparison, was not different among CRC, adenoma, HV, and control groups.

It was found that both CRC and adenoma patients showed significantly higher *S. gallolyticus *IgG antibodies than control group. Mean ELISA readings in terms of optical density (OD) were used to measure the serum level of *S. gallolyticus *IgG antibodies in both CRC (0.158 ± 0.032), and adenoma patients (0.173 ± 0.024) which were higher than that of HV group (0.064 ± 0.011) and control subjects (0.046 ± 0.024) (P < 0.05). However, there was no significant difference between the serum level of *S. gallolyticus *IgG antibodies of CRC and of adenoma patients (P > 0.05) or between the serum level of *S. gallolyticus *IgG antibodies of HV group and of control group (P > 0.05) (figure 1). On the other hand, it was found that the seroprevalence of *B. fragilis*, as an indicator for any access of the large intestine flora into the circulation, was not higher in CRC patients (0.166 ± 0.013) and colorectal adenoma patients (0.178 ± 0.032) than in HV members (0.176 ± 0.014) and control group (0.18 ± 0.02) (P > 0.05) (figure 1). In addition, there was no significant difference found in the serum level of *S. gallolyticus *IgG antibodies among CRC patients in terms of age, sex, and tumor site, colon versus rectum, and colon segments (P > 0.05).

**Figure 2:**
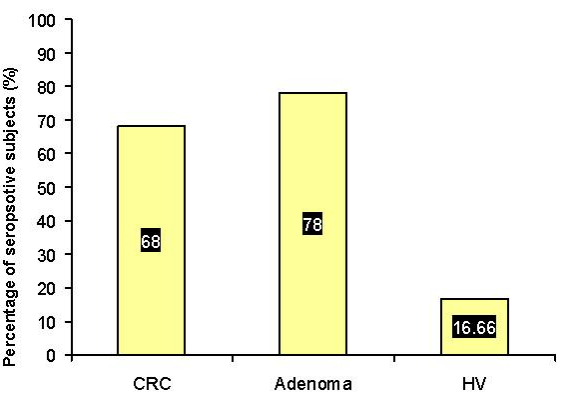
**The percentage of seropositives for S. gallolyticus IgG antibodies in CRC, adenoma, and HV groups depending on the cutoff value calculated from the tumor-free control group**. Colorectal adenoma and CRC groups showed high percentage of *S. gallolyticus *IgG seropositives while HV group showed much lower percentage.

The cutoff value for *S. gallolyticus *seroprevalence, which was calculated from the control group, was equal to OD value of 0.07. By applying this cutoff value on CRC, adenoma, and HV groups, it was found that the percentage of seropositive subjects for *S. gallolyticus *IgG antibodies in CRC group, 34 out of 50 (68%), was close to that of adenoma group, 11 out of 14 (78%) (P > 0.05). However, these percentages were remarkably higher than that of HV group, 5 out of 30 (16.66%) (P < 0.05) (figure 2). Accordingly, the use of this cutoff value in ELISA for the seropositivity of *S. gallolyticus *IgG antibodies yielded sensitivity of 68% for CRC detection and 78% for adenoma detection and specificity of 83.33%. There was no significant difference in age and sex between seropositive and seronegative groups of CRC, adenoma, and HV groups (P > 0.05). The 5 seropositive members of HV group were traced and 4 of them volunteered to do colonoscopy. Three members showed normal colonic mucosa and one showed begnine hyperplastic polyp (0.7 cm in diameter) in the distal colon. There was no particular history of CRC and colorectal adenoma in the seropositive members of HV group.

### In situ hybridization expression of NF-κB mRNA and IL-8 mRNA in CRC and adenoma patients

#### ISH immunostaining of mucosal (adeno) cells

**Figure 3:**
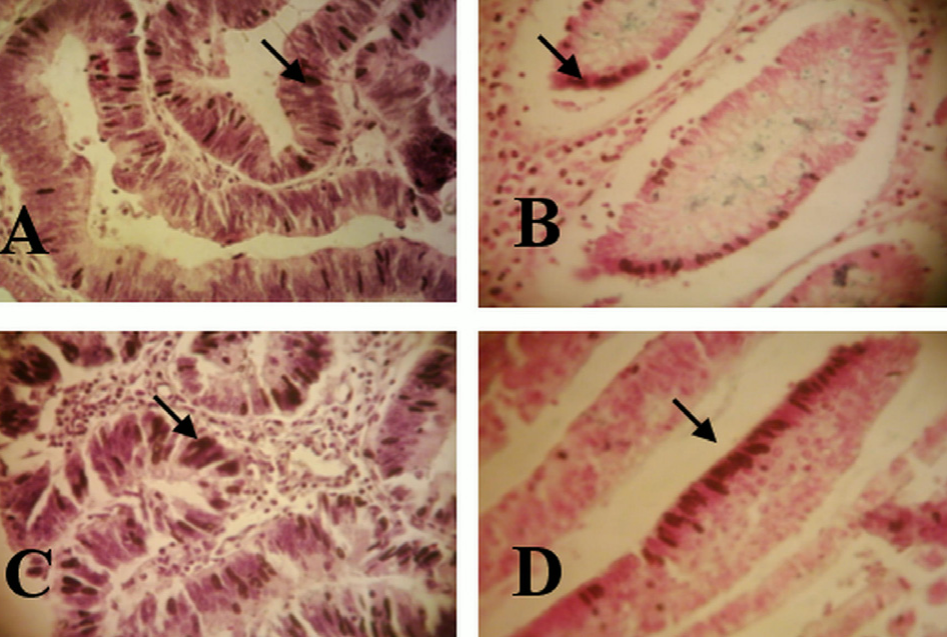
**Examples for NF-κB mRNA expression in the tumorous sections of CRC patients**. (A) positive tissue section, >50% stained cells, and (B) tissue section, 5%-25% stained cells with stained stromal cells. Examples for IL-8 mRNA expression in CRC patients at (C) score 2, 11%-50% stained cells and at (D) score 1, 1%-10% stained cells. Cells stained positive are shown with dark color of NBT/BCIP in the nuclei of glandular cells (adenocarcinoma cells) (×400) and pointed by black bold arrow.

The expression level of NF-κB mRNA and IL-8 mRNA (figure 3) was tested by ISH staining in tumorous versus non-tumorous tissue sections in CRC and adenoma patients (Table 1 and 2) and normal mucosal tissue sections from control subjects versus tumorous and non-tumorous tissue sections of CRC and adenoma patients (Table 1 and 2). In addition, the mRNA expression of NF-κB and IL-8 was tested in tumorous and non-tumorous tissue sections of CRC versus that of adenoma patients (Tables 3 and 4) and in CRC *S. gallolyticus *seropositives (CRC-Sg+ve) versus that of CRC *S. gallolyticus *seronegatives (CRC-Sg-ve) (Tables 3 and 4).

**Table 1:** The expression of NF-κB mRNA in the tumorous and the non-tumorous tissue sections of CRC and adenoma patients as well as in normal tissue sections of control subjects.

Tissue sections of NF-κB	Positive NF-κB mRNA expression N (%)	χ^2 ^test *P *value	Mean % of NFKB-positively stained cells	P value for positively stained cells
**CRC:**				
Tumorous	36/50 (72)	0.0006	74.7 ± 4.7	<0.01
Non-tumorous	19/50 (38)		36.23 ± 3.6	
				
Tumorous	36/50 (72)	<0.0001	74.7 ± 4.7	<0.01
Normal tissue	4/30 (13.3)		21.8 ± 2.3	
				
Non-tumorous	19/50 (38)	0.018	36.23 ± 3.6	<0.01
Normal tissue	4/30 (13.3)		21.8 ± 2.3	
				
**Adenoma:**				
Tumorous	7/14 (50)	0.04	57.73 ± 5.6	<0.01
Non-tumorous	2/14 (14.2)		27.85 ± 5.8	
				
Tumorous	7/14 (50)	0.009	57.73 ± 5.6	<0.01
Normal tissue	4/30 (13.3)		21.8 ± 2.3	
				
Non-tumorous	2/14 (14.2)	0.9	27.85 ± 5.8	>0.05
Normal tissue	4/30 (13.3)		21.8 ± 2.3	

**Table 2:** The expression of IL-8 mRNA in the tumorous and the non-tumorous tissue sections of CRC and adenoma patients as well as in normal tissue sections of control subjects.

Tissue sections of IL-8	Median score of IL-8 mRNA expression	P value for Mann-Whitney of ordinal scores	Mean percentage of IL-8 mRNA positively stained cells (%)	P value for positively stained cells
**CRC:**				
Tumorous	Score 2	0.018	26.6 ± 2.05	<0.01
Non-tumorous	Score 1		9.28 ± 1.3	
				
Tumorous	Score 2	0.018	26.6 ± 2.05	<0.01
Normal tissue	Score 1		7.3 ± 1.44	
				
Non-tumorous	Score 1	0.56	9.28 ± 1.3	>0.05
Normal tissue	Score 1		7.3 ± 1.44	
				
**Adenoma:**				
Tumorous	Score 2	0.018	23.71 ± 1.86	<0.01
Non-tumorous	Score 1		7.92 ± 1.73	
				
Tumorous	Score 2	0.018	23.71 ± 1.86	<0.01
Normal tissue	Score 1		7.3 ± 1.44	
				
Non-tumorous	Score 1	0.56	7.92 ± 1.73	>0.05
Normal tissue	Score 1		7.3 ± 1.44	

**Table 3:** The expression of NF-κB mRNA in the tumorous and non-tumorous tissue sections of CRC versus adenoma patients and in CRC-Sg+ve versus CRC-Sg-ve groups.

tissue sections	Positive NF-κB mRNA expression N (%)	χ^2 ^test *P *value	Mean % of NFKB-positively stained cells	P value for positively stained cells
**Tumorous tissue sections**				
**Patients:**				
CRC	36/50 (72)	0.042	74.7 ± 4.7	<0.01
Adenoma	7/14 (50)		57.73 ± 5.6	
				
***S. gallolyticus *seropositivity**				
CRC-Sg+ve	28/34 (82)	0.017	79.45 ± 6.2	<0.05
CRC-Sg-ve	8/16 (50)		64.6 ± 4.6	
				
**Non-tumorous tissue sections**				
**Patients:**				
CRC	19/50 (38)	0.09	36.23 ± 3.6	>0.05
Adenoma	2/14 (14.2)		27.85 ± 5.8	
				
***S. gallolyticus *seropositivity**				
CRC-Sg+ve	17/34 (50)	0.01	40.25 ± 3.7	<0.05
CRC-Sg-ve	2/16 (12.5)		27.7 ± 4.2	

**Table 4:** The expression of IL-8 mRNA in the tumorous and non-tumorous tissue sections of CRC versus adenoma patients, and in CRC-Sg+ve versus CRC-Sg-ve groups.

Tissue sections	Median score of IL-8 mRNA expression	Mann-Whitney for median scores (P value)	Mean percentage of IL-8 mRNA positively stained cells (%)	student *t *test for positively stained cells (P value)
**Tumorous tissue sections**				
**Patients:**				
CRC	Score 2	0.64	26.6 ± 2.05	>0.05
Adenoma	Score 2		23.7 ± 1.86	
				
***S. gallolyticus *seropositivity:**				
CRC-Sg+ve	Score 2	0.049	31.58 ± 4.2	<0.01
CRC-Sg-ve	Score 1.5		16 ± 2.8	
				
**Non-tumorous tissue sections**				
**Patients:**				
CRC	Score 1	0.56	9.28 ± 1.3	>0.05
Adenoma	Score 1		7.92 ± 1.73	
				
***S. gallolyticus*** **seropositivity:**				
CRC-Sg+ve	Score 1	0.56	10 ± 2.9	>0.05
CRC-Sg-ve	Score 1		7.56 ± 1.7	

At first, it was important to explore whether the mRNA expression of NF-κB and IL-8 is intensified in tumor cells of CRC and adenoma in comparison with normal mucosal tissues of control subjects and the surrounding mucosal tissue (non-tumorous) or not. It was found that the mRNA expression of NF-κB and IL-8 in the tumorous tissue sections of CRC and adenoma patients was higher than in the normal mucosal tissue sections of the control subjects and that of the corresponding non-tumorous tissue sections (P < 0.05) (Table 1 and 2). This indicated a significant association of the expression of NF-κB mRNA and IL-8 mRNA with the tumorous tissues of both CRC and adenoma patients rather than the surrounding non-tumorous tissues or normal mucosal tissues. Moreover, the non-tumorous tissue sections of CRC patients but not adenoma patients showed higher levels of expression of NF-κB mRNA but not IL-8 mRNA than that of normal mucosal tissues of control subjects (Table 1). This provided evidence that NF-κB mRNA expression, unlike IL-8, increases in the tumor-adjacent normal mucosal cells in patients with CRC but not adenoma.

The NF-κB mRNA expression in tumorous tissue sections of CRC patients was associated with the seropositivity of *S. gallolyticus *IgG antibodies in that its expression was higher in CRC-Sg+ve group than in CRC-Sg-ve group (P < 0.05). And NF-κB mRNA expression was higher in carcinoma than in adenoma patients (P < 0.05) (Table 3). However, it was found that, just like the tumorous tissue sections, the expression of NF-κB mRNA in non-tumorous tissue sections was higher in CRC-Sg+ve group than in CRC-Sg-ve group (P < 0.05) but was not higher in CRC than in adenoma (P > 0.05) (Table 3). This indicated that high levels of NF-κB mRNA expression were associated significantly with the seropositivity of *S. gallolyticus *IgG antibodies in both tumorous cells and histologically normal adjacent cells making NF-κB as a remarkable factor associated with the hypothesis of *S. gallolyticus-*associated/triggered CRC. Moreover, NF-κB was shown to be associated with the transformation process from adenoma to CRC. On the other hand, the level of NF-κB mRNA expression in CRC patients was not related with staging or with tumor grade (P > 0.05). It is noteworthy to mention that the positive expression of NF-κB was 0% in negative control tissue section, and 73% in positive control tissue section.

For IL-8 mRNA expression, the negative control sections showed score 0 and the positive control section showed score 2. In tumorous tissue sections, IL-8 mRNA expression was associated with the seropositivity of *S. gallolyticus *IgG antibodies in that its expression was higher in CRC-Sg+ve group than in CRC-Sg-ve group (P < 0.05). On the other hand, there was no significant difference between IL-8 mRNA expression in CRC versus adenoma patients (P > 0.05) (Table 4). In the non-tumorous tissue sections, it was found that the positive expression of IL-8 mRNA was not higher in CRC-Sg+ve group than in CRC-Sg-ve group (P > 0.05) nor was higher in CRC than in adenoma (P > 0.05) (Table 4). This indicated that IL-8, unlike NF-κB, was not much associated with the transformation process of adenoma to CRC but, like NF-κB, was significantly associated with *S. gallolyticus *in CRC patients.

By means of Pearson's correlation, it was shown that the higher the level of *S. gallolyticus *IgG antibodies represented by ELISA readings, the higher the expression of IL-8 mRNA in the tumorous tissue sections of CRC patients; the correlation was significant and positive (*r *= +0.32, P < 0.05). Moreover, higher expression of IL-8 mRNA in CRC was associated with more advanced stages of CRC (P < 0.05) but not with tumor grade (P > 0.05), in that CRC patients who showed high IL-8 mRNA expression (scores 2-3) were 3/8 (37.5%) of stage B, 6/12 (50%) of stage C, and 24/30 (80%) of stage D.

It is noteworthy to mention that the level of expression of tumor suppressor proteins p21, p27 and p53 which were examined previously on the same samples by Immunohistochemistry assay, [unpublished data] showed no association with the seropositivity of *S. gallolyticus *in CRC patients.

#### ISH immunostaining of stromal cells

IL-8 and NF-κB mRNA immunostaining of stromal cells rather than glandular mucosal cells (adeno cells) showed much lower levels than that of glandular mucosal cells in both tumorous and non-tumorous tissue sections of CRC and adenoma patients (P < 0.05) (Table 5). The mRNA expression of IL-8 and NF-κB in stromal cells of CRC but not of adenoma patients was borderline higher in tumorous than both normal and non-tumorous tissue sections (P < 0.05). However, there was no difference between non-tumorous and normal tissue sections in both CRC and adenoma patients (P > 0.05) (Table 5). IL-8 mRNA and NF-κB mRNA expression in stromal cells of both tumorous and non-tumorous tissue sections was not different between CRC and adenoma patients and between CRC-Sg+ve and CRC-Sg-ve groups (P > 0.05) (Table 5). This indicated that mRNA expression of IL-8 and NF-κB in stromal cells, like glandular cells, showed association with CRC tumorous cells in comparison with non-tumorous and normal cells, however, unlike glandular cells, it showed no association with adenoma, the transformation process from adenoma to CRC, and no association with the seropositivity of *S. gallolyticus *IgG antibodies. This might be attributed to the fact that the involved CRC cases in this study were all of adenocarcinoma type.

**Table 5:** The expression of NF-κB and IL-8 mRNA in the stromal cells of the tumorous and non-tumorous tissue sections of CRC, adenoma, CRC-Sg+ve, and CRC-Sg-ve groups.

Tissue section	Mean percentage of NF-κB mRNA expression %	Mean percentage of IL-8 mRNA expression %
Normal tissue	21.8 ± 2.3	7.3 ± 1.44
Tumorous CRC	29.92 ± 3.5	11.55 ± 1.07
Non-tumorous CRC	23.2 ± 2.8	7.1 ± 1.3
Tumorous adenoma	24.15 ± 2.7	9.5 ± 1.37
Non- tumorous adenoma	23.06 ± 1.6	8.4 ± 1.1
Tumorous CRC-Sg+ve	30.41 ± 2.3	12.36 ± 1.72
Ttumorous CRC-Sg-ve	28.88 ± 1.85	9.84 ± 1.25
Non-tumorous CRC-Sg+ve	22.5 ± 1.74	7.2 ± 1.21
Non-tumorous CRC-Sg-ve	24.7 ± 1.52	6.8 ± 0.62

## Discussion

There are several microbial candidates, other than *S. gallolyticus*, for the association with CRC such as *S. pasterianus*, *S. infantarius*, *S. salivarius, H. pylori*, and some strains of *E. coli *[2,3,7,9,17]. However, except for *H. pylori*, the association of these bacteria has not been found as obvious as *S. gallolyticus*. Nevertheless, few studies have been conducted to investigate the serological aspect of *S. bovis *association with colorectal carcinoma and adenoma and no study has been conducted to investigate the serological association of *S. gallolyticus *with colorectal carcinoma and adenoma. The design of ELISA of the current study took into account the feasibility of measuring IgG antibodies towards *S. gallolyticus *and *B. fragilis *rather than IgM due to the expected long period of exposure to these bacteria, if any. It was previously shown that titers of IgM antibodies of *S. bovis *biotype I in CRC patients lack the consistency because the immune stimulation of CRC patients towards *S. bovis *has occurred over a long period of time [26].

In this study, the level of *S. gallolyticus *IgG antibodies was significantly higher in CRC and adenoma patients than in control and HV groups. Darjee and Gibb stated that patients with colonic cancer had higher median IgG antibody titers to *S. bovis *and *E. faecalis *preparations than control samples did [27]. Hence, the seroprevalence of IgG antibodies against *S. gallolyticus *subspecies *S. gallolyticus*, measured in the current study, showed the same behavior of the seroprevalence of IgG antibody against *S. bovis *NCTC 8133 (now is termed *S. infantiarus*). Moreover, the findings of the study of Devriese et al support our findings. They stated that *S. gallolyticus *is more likely to be involved in human infections which and associated with colonic cancer [8].

The association of seroprevalence of *S. gallolyticus *with CRC and colorectal adenoma in this study was specific because of the lack of similar seroprevalence association of the other studied bacteria, namely *B. fragilis*. *B. fragilis *was selected as one of the best candidate indicators for monitoring any history of breach of the mucosal barrier between large intestine and blood circulation by ulceration, tumor lesions, surgery, or necrosis. This draws our attention on how *S. gallolyticus *could be associated with CRC and adenoma. Is this association just a consequence of the tumor lesion or this bacterium might act as tumor initiator or promoter. Wanke and Bistrian revealed that local actions of cytokines or of chemical mediators able to promote vasodilatation and the enhancement of capillary permeability, may support bacterial entry at the tumor site, and increase bacterial adherence to various cells [28]. If this scenario was the explanation for the higher seroprevalence of *S. gallolyticus *in CRC and adenoma patients than in HV and control groups, we should have seen the same association for *B. fragilis *which is by far more dominant than *S. gallolyticus *in the bowel and of 100% incidence.

In addition, the association of *S. gallolyticus *with adenomatous polyps was another clue supporting the hypothesis of the association of *S. gallolyticus *with the transformation of colorectal mucosa from early adenomatous polyp stages to late CRC stages, taken into account that 90% of preinvasive neoplastic lesions of the colorectum are polyps or polyp precursors (aberrant crypt foci) [29]. The findings of this study were in agreement with other studies like Fagundes et al. who revealed that this bacterium among patients with septicemia and/or endocarditis is also related, in a significant way, to the presence of villous or tubulovillous adenomas in the large intestine [6]. Therefore, the serological investigation of *S. gallolyticus *in this study has shown 4 factors of reliability; the statistical significance of *S. gallolyticus *association with both adenomas and CRC, no similar association was seen by the indicator bacteria *B. fragilis*, relying on the measurement of IgG rather than IgM antibodies which reflects better the status of longstanding immune reaction against bacterial antigens, and the agreement of our results with other studies that based on isolating *S. gallolyticus *from blood at the bacteremic phase.

A colonoscopically proven control group was involved in order to obtain punch biopsies of normal mucosal tissues and also to calculate precisely the cutoff value of normal seropositivity of *S. gallolyticus *IgG antibodies. After applying this cutoff value on HV, CRC, and adenoma groups, it was apparent that such cutoff value can be used to screen the high risk groups of the population at sensitivity ranges from 68 to 78% for CRC and adenoma and at specificity of 83.33%. Therefore, this assay can be used in designing an optimized kit for screening and/or detecting CRC or colorectal adenoma in the population by using simple, cheap and noninvasive procedure. Four seropositive subjects in HV group were examined by colonoscopy, 3 were normal and 1 revealed a medium-sized non-malignant hyperplasic polyp. The detected hyperplasic polyp might be an earlier event than adenoma for the association of *S. gallolyticus *with transformation of colonic mucosa. However, this needs a prospective study on an extensive population to determine on solid basis the association extent of benign polyps with *S. gallolyticus*.

The mRNA expression of NF-κB and IL-8 was significantly higher in tumor cells than in histologically normal adjacent cells in both CRC and adenoma patients and it was higher in CRC and adenoma than in control subjects. This provided evidence that NF-κB and IL-8 might have a remarkable role in the transformation of colorectal adenoma and CRC. Several steps were dedicated to explore the nature of the association of NF-κB and IL-8 with colorectal adenoma and CRC in terms of relationship with *S. gallolyticus*. It was found that the expression of NF-κB mRNA in CRC-Sg+ve was significantly higher than in CRC-Sg-ve patients in both tumorous and non-tumorous tissue sections, and was higher in CRC than in adenoma patients in tumorous rather than non-tumorous tissue sections. These results were of high value by the virtue of many reasons. First, to the best of our knowledge, no previous reports were found to elucidate any possible relationship between NF-κB and *S. gallolyticus *in CRC and adenoma patients. Second, these findings pinpoint to a possibility that NF-κB could be an essential factor for the carcinogenic effect of *S. gallolyticus *on colorectal mucosa. NF-κB is implicated in the transcription of many target genes that exert carcinogenic role and many studies revealed that the elevation of NF-κB activity was evident in a number of human cancers, including breast cancer [30], thyroid cancer [31], melanoma [32], and colon cancer [33,34]. Third, NF-κB mRNA expression was significantly higher in CRC than in adenoma patients indicating that NF-κB might play an important role in the transformation sequel from normal mucosa, to adenoma, and to malignant CRC stage, where NF-κB expression was highest.

The probable mechanism of carcinogenesis by *S. gallolyticus *via NF-κB is believed to be driven by the downstream mediators of NF-κB. It was revealed that the synthesis of many cytokines and active compounds, which are transcriptionally dependent on NF-κB, is in response to *S. bovis *and leads to the formation of COX-2, nitric oxide, and free radicals such as superoxide [35], peroxynitrites, and hydroxyl radicals [36], which all possess a highly potent mutagenicity. Furthermore, *S. bovis *was proved experimentally to induce *ras *oncogene [3] which requires the cell survival function of NF-κB to overcome death signal initiated in transformed cells [37]. Therefore, like *S. bovis*, it is possible that *S. gallolyticus *uses NF-κB as a survival tool against death signals along with its potentially mutagenic downstream mediators.

Regarding IL-8, its mRNA expression in tumorous rather than non-tumorous tissue sections of CRC-Sg+ve was significantly higher than in CRC-Sg-ve patients. Nevertheless, IL-8 mRNA expression in both tumorous and non-tumorous tissue sections of CRC was not higher than that of adenoma patients. Pearson's correlation coefficient revealed that the level of *S. gallolyticus *IgG antibodies was positively correlated with IL-8 mRNA expression (*r*+0.32, P = 0.022) indicating that *S. gallolyticus *seroprevalence might be in parallel with IL-8 mRNA expression in CRC. Furthermore, higher expression of IL-8 mRNA in CRC was associated significantly with more advanced stages of CRC. These findings highlighted several interpretations. First, IL-8 is most probably associated with *S. gallolyticus *and correlated well with its seroprevalence level which might indicate a role to play for the association between this bacterium and CRC. Second, unlike NF-κB, IL-8 association with *S. gallolyticus *was restricted to tumorous rather than non-tumorous cells which might indicate a propagating rather than promoting oncogenic role of *S. gallolyticus *via IL-8. Third, in supporting the earlier analysis, the level of IL-8 mRNA was not significantly different between adenoma and CRC patients in both tumorous and non-tumorous tissue sections while it was clearly associated with disease progression and CRC staging.

The pattern of IL-8 mRNA expression led us to propose that IL-8 is more likely a propagating factor for CRC and lacks the promoting role in the transformation process from colorectal adenoma to CRC. This might be attributed to the angiogenic role of IL-8 by which new blood vessels are formed to meet the increasing demands of cancer growth. These findings are supported by Ellmerich et al. who revealed that small amounts of IL-8 were detected in the mucosa of control rats, while the level of IL-8 was ~4 to 3 folds higher in the mucosa of rats receiving *S. bovis *and *S. bovis *wall extracted antigen fraction, respectively [38]. On the other hand, because IL-8 transcription was shown to be dependent on the cooperative action of two transcription factors, NF-κB and AP-1 [39], hence, both NF-κB and IL-8 might play an integrated role leading to a series of steps to escape from death signals, release of potentially mutagenic products, and preparing the needed blood supply to the cancerous cells. Regarding the ISH staining of stromal cells, they were found to express mRNA of NF-κB and IL-8 much lower than that of glandular mucosal cells. Although mRNA expression of NF-κB and IL-8 was not associated in stromal cells with *S. gallolyticus *IgG antibodies, the NF-κB and IL-8 expression was higher in tumorous CRC than in tumorous adenoma sections. Accordingly, glandular rather than stromal cells have shown to be mainly responsible for the expression of NF-κB and IL-8 as well as glandular rather than stromal cells seem to play the major role in the association of *S. gallolyticus *with colonic mucosa.

## Conclusion

Taken together, *S. gallolyticus *subspecies *gallolyticus *was found serologically associated with both CRC and colorectal adenoma. The seroprevalence of *S. gallolyticus *IgG antibodies appeared as a reliable indication for the association of this bacterium with CRC and adenoma. Moreover, *S. gallolyticus *has shown a specific association with CRC and colorectal adenoma when compared with the more dominant intestinal bacteria, *B. fragilis*. This provided evidence for a possible important role of *S. gallolyticus *in the carcinogenesis of CRC from pre-malignant polyps. In addition, the seroprevalence of *S. gallolyticus *has shown to be a good candidate for formulating a screening assay for the early detection of both adenoma and CRC cases in high risk groups. Regarding the possible underlying mechanisms of such association, NF-κB and IL-8 rather than other transformation factors p21, p27 and p53 [unpublished data] might highly act as important mediators for the *S. gallolyticus*-associated carcinogenesis of adenoma to carcinoma. Moreover, it was concluded that NF-κB exerts most probably a promoting carcinogenic effect and IL-8 exerts mainly a propagating effect on colorectal mucosal cells. We recommend conducting further studies to reveal the in vitro effect of *S. gallolyticus *on colorectal mucosal cells in relation to factors such as COX-2, Ras, apoptosis markers, PGE2, TNF-alpha, IL-1, and IL-6. We also recommend conducting a prospective screening study on the high risk groups of the population using the formulated ELISA kit to measure the seropositivity of *S. gallolyticus *IgG antibodies and assess the reliability of such screening assay when conducted on a large scale sample of the population.

## Competing interests

The authors declare that they have no competing interests.

## Authors' contributions

AS carried out the sampling, taking the medical history, patients examination, and supervision on the immunological tests. AS, RR, and LK carried out the bacteriological cultures, cell wall extraction, and LPS extraction. T and LK supervised the immunological testing and edited the outline of the manuscript. AS, RR and F carried out the pathological and histological examination, the classification of the biopsies, and ISH. AS, RR, and T carried out the statistical design, statistical analysis, and the design of the cut off value. All authors read and approved the final manuscript.

## Pre-publication history

The pre-publication history for this paper can be accessed here:

http://www.biomedcentral.com/1471-2407/9/403/prepub
